# Comprehensive analysis of cuproptosis-related long noncoding RNA for predicting prognostic and diagnostic value and immune landscape in colorectal adenocarcinoma

**DOI:** 10.1186/s40246-023-00469-5

**Published:** 2023-03-13

**Authors:** Shichao Liu, Shoucai Zhang, Yingjie Liu, XiaoRong Yang, Guixi Zheng

**Affiliations:** 1 Department of Clinical Laboratory, Qilu Hospital of Shandong University, Jinan, China; 2 Department of Clinical Laboratory, Qilu Hospital, Cheeloo College of Medicine, Shandong University, Jinan, 250012 Shandong Province People’s Republic of China; 3 Shandong Engineering Research Center of Biomarker and Artificial Intelligence Application, Jinan, China; 4 Clinical Epidemiology Unit, Qilu Hospital of Shandong University, Jinan, China

**Keywords:** Cuproptosis, Long noncoding RNA, Colon adenocarcinoma, Prognosis, Diagnosis, Immune microenvironment

## Abstract

**Background:**

Cuproptosis, as a copper-induced mitochondrial cell death, has attracted extensive attention recently, especially in cancer. Although some key regulatory genes have been identified in cuproptosis, the related lncRNAs have not been further studied. Exploring the prognostic and diagnostic value of cuproptosis-related lncRNAs (CRLs) in colon adenocarcinoma and providing guidance for individualized immunotherapy for patients are of great significance.

**Results:**

A total of 2003 lncRNAs were correlated with cuproptosis genes and considered as CRLs. We screened 33 survival-associated CRLs and established a prognostic signature base on 7 CRLs in the training group. The patients in the low-risk group had better outcomes in both training group (*P* < 0.001) and test group (*P* = 0.016). More exciting, our model showed good prognosis prediction in both stage I–II (*P* = 0.020) and stage III–IV (*P* = 0.001). The nomogram model could further improve the accuracy of prognosis prediction. Interestingly, glucose-related metabolic pathways, which were closely related to cuproptosis, were enriched in the low-risk group. Meanwhile, the immune infiltration scores were lower in the high-risk group. The high-risk group was more sensitive to OSI.906 and ABT.888, while low-risk group was more sensitive to Sorafenib. Three lncRNAs, FALEC, AC083967.1 and AC010997.4, were highly expressed in serum of COAD patients, and the AUC was 0.772, 0.726 and 0.714, respectively, indicating their valuable diagnostic value.

**Conclusions:**

Our research constructed a prognostic signature based on 7 CRLs and found three promising diagnostic markers for COAD patients. Our results provided a reference to the personalized immunotherapy strategies.

**Supplementary Information:**

The online version contains supplementary material available at 10.1186/s40246-023-00469-5.

## Introduction

As one of the most common malignancies, colorectal cancer (CRC) is the second leading cause of cancer-related death [1]. According to the analysis of global cancer statistics in 2020, there were 1.9 million new cases and 935,000 deaths of CRC, accounting for about one-tenth of all malignant tumors [2]. CRC includes colon cancer (CC) and rectal cancer (RC), among which colorectal adenocarcinoma (COAD) is the main type. In addition to surgery, targeted therapy, chemotherapy and immunotherapy are common treatments for COAD, and approximately two-thirds of patients with stage III that receive adjuvant chemotherapy can reduce the risk of recurrence [3]. In recent years, targeted therapy and immunotherapy for COAD have also made great progress [4–9]. Therefore, it is particularly important to provide viable biomarkers for prognosis prediction and personalized treatment for COAD patients.

Cuproptosis, a novel cell death type, which is caused by the accumulation of intracellular copper triggering the aggregation of mitochondrial lipoylated proteins and the destabilization of Fe–S cluster proteins [10]. Because mitochondria are the main place of glycolysis, which is very important for the proliferation of cancer cells. Therefore, the malignant potential of tumor cells can be reduced by regulating cuproptosis to inhibit glucose metabolism [11]. Besides, the therapeutic effect of copper and its complex has also been confirmed in cancer [12–14], which includes inducing autophagic cell death by targeting ULK1 in colorectal cancer [15], causing immunogenic cell death of breast cancer stem cells [16] and leading to caspase-independent cell death about diffuse large B cell lymphoma [17]. Thus, it may be meaningful to explore the impact of cuproptosis on tumor microenvironment (TME) and cancer therapy.

Long noncoding RNAs (lncRNAs) with transcription length of more than 200 nucleotides are transcribed by RNA polymerase II [18]. Although there is no potential to encode proteins, lncRNAs play vital roles in tumorigenesis and metastasis through gene transcription and post transcriptional modification [19–21]. LncRNAs are considered as promising biomarkers for early-stage detection, diagnosis, prognosis and prediction of drug therapy response in cancers [22, 23], such as lung cancer [24], gastric cancer [25], liver cancer [26], breast cancer [27], colorectal cancer [28] and so on. In addition, studies have shown that the extraction of lncRNAs is related to glucose metabolism tumors [29]. However, whether cuproptosis-related lncRNAs (CRLs) play important roles in COAD has not been explored.

In our study, we analyzed the expression of 18 cuproptosis-related genes (CRGs) to screen the related lncRNAs. The differentially expressed CRLs between COAD tumors and normal tissues were analyzed. All samples were randomly divided into training and test groups at the ratio of 7:3. By univariate Cox regression analysis, 33 survival-associated CRLs were identified in training group. Following, we enrolled 7 CRLs to establish a prognostic model by multivariate Cox regression analysis. The COAD patients were divided into high-risk and low-risk groups according to risk score. The Kaplan–Meier (K–M) survival curves of training and test groups both showed the low-risk group had better outcomes. The 1-, 2-, 3-year ROC curves also verified its accuracy of prognostic prediction. Besides, we constructed a nomogram model based on independent risk factors, including risk score, age and T stage which had more excellent prognosis prediction ability. We also performed functional enrichment analysis of high-risk and low-risk groups by Gene Set Enrichment Analysis (GSEA) software. The correlation of risk score with clinical parameters was also analyzed. Furthermore, the differences of immune cells scores, immune functions scores, immune checkpoints and drug treatment response between two risk groups were explored. Finally, we explored the diagnostic value of CRLs in serum. Our results provided a promising direction for the study of cuproptosis-related lncRNAs in COAD and contributed to the development of personalized immunotherapy for COAD patients.

## Materials and methods

### Data acquisition and analysis

The transcriptomic data of 473 COAD tumors and 41 normal samples were downloaded from the TCGA database [30]. Then, we separated the expression of 14,056 lncRNAs and 19,573 mRNAs in COAD samples by Strawberry Perl. The clinical information of 421 COAD patients was also obtained from TCGA database after excluding samples with short-term survival (less than 30 days) or missing follow-up days (Table 1). By data merging, 417 COAD patients were finally included in the present analysis. We collected 18 cuproptosis-related mRNAs from previous literature [31–36], including FDX1, DLD, PDHA1, PDHB, MTF1, GLS, CDKN2A, DLAT, LIAS, LIPT1, LIPT2, ATP7A, ATP7B, SLC31A1, SLC31A2, DLST, NFE2L2, NLRP3 and extracted the expression of those CRGs from COAD samples accordingly.

**Table 1:** The clinical characteristics of COAD patients

Characteristics		Samples	Percent (%)
Gender	Female	179	45.3
	Male	216	54.7
Age	≤ 60y	119	30.1
	> 60y	276	69.9
Clinical stages	Stage I	67	17
	Stage II	153	38.7
	Stage III	113	28.6
	Stage IV	51	12.9
	Unknown	11	2.8
T stages	T0/Tis	1	0.3
	T1	9	2.3
	T2	68	17.2
	T3	269	68.1
	T4	48	12.2
N stages	N0	233	59
	N1	95	24
	N2	67	17
M stages	M0	297	75.2
	M1	51	12.9
	Unknown	47	11.9
Survival status	Alive	323	81.8
	Dead	72	18.2
Treatment or therapy	Yes	67	17
	No	296	74.9
	Unknown	32	8.1

Serum of 150 COAD patients and 135 healthy controls was collected. The diagnosis of COAD patients was confirmed by histopathology or biopsy and recruited from the Department of General Surgery, Qilu Hospital of Shandong University, from April 2018 to October 2020. The healthy controls were enrolled from the Department of Physical Examination Center, Qilu Hospital of Shandong University. Serum samples were separated by centrifugation at 6000 g for 10 min followed by another centrifugation at 12,000 g for 10 min and then stored at − 80 °C for further analysis.

### Identification of differentially expressed cuproptosis-related LncRNAs

After the Pearson correlation algorithm with the filter of |coefficient|> 0.3 and *P* < 0.001, we selected lncRNAs that were related with cuproptosis genes and considered as CRLs. Next, we identified differentially expressed lncRNAs (|Log_2_ fold change (FC)|> 1, false discovery rate (FDR) < 0.05) in COAD tumor tissues comparing with normal tissue using differential analysis by R package “limma” [37].

### Establishment and evaluation of CRLs prognostic signature

After the COAD patients were randomly divided into training and test groups, we first performed univariate Cox analysis (*P* < 0*.*01) to screen CRLs associated with prognosis. Next, we established a prognostic signature by multivariate Cox regression analysis in the training group. Hence, the risk score of each COAD patient could be calculated according to the coefficient and CRLs expression in our prognostic signature. And the COAD patients were divided into the high-risk and low-risk groups by the median value of the risk score.

We used the Kaplan–Meier (K–M) and receiver operating characteristic (ROC) curves to evaluate the value of the prognostic signature in both training and test groups by R packages “survival,” “survminer” and “timeROC” [38]. Besides, the relationship between the risk score and prognosis of COAD patients was also displayed by heatmap jointly, risk score curve, and survival status diagram. Moreover, whether the risk score was related with clinical parameters was also examined.

### Independent prognostic analysis and development of nomogram model

The univariate and multivariate Cox regression analyses were performed to identify the independent risk factors of COAD, including risk score and clinical characteristics. Subsequently, the nomogram model was constructed based on independent risk factors using the R package “rms.” Then, we used calibration curves to estimate the prediction power of the model.

### Gene enrichment analysis by GSEA

To identify pathway enrichment in two risk groups, we used GSEA software (4.2.2) to perform the enrichment analysis of the Kyoto Encyclopedia of Genes and Genomes (KEGG) (c2.cp.kegg.v7.5.1.symbols.gmt) [39]. The random sample permutation number was set as 1,000, and the significance threshold was *P* < 0.05.

### Immune infiltration analysis by single sample gene set enrichment analysis (ssGSEA)

The enrichment score of infiltration estimation and immune function of different immune cells between two risk groups was compared using ssGSEA analysis [40] by R packages “GSVA,” “GSEABase” and “Limma.” So, we could easily explore the association between risk score, immune infiltration and immune function. And the significance threshold was FDR < 0.05.

### The value of risk score in predicting response of patients to immunotherapy and chemotherapy

We also analyzed the differential expression of 47 immune checkpoint genes, including IDO1, LAG3, CTLA4, TNFRSF9, ICOS, CD80, PDCD1LG2, TIGIT, CD70, TNFSF9, ICOSLG, KIR3DL1, CD86, PDCD1 (PD1), LAIR1, TNFRSF8, TNFSF15, TNFRSF14, IDO2, CD276, CD40, TNFRSF4, TNFSF14, HHLA2, CD244, CD274 (PD-L1), HAVCR2, CD27, BTLA, LGALS9, TMIGD2, CD28, CD48, TNFRSF25, CD40LG, ADORA2A, VTCN1, CD160, CD44, TNFSF18, TNFRSF18, BTNL2, C10orf54, CD200R1, TNFSF4, CD200, NRP1 between two risk groups. Besides, we collected 15 commonly used drugs for the clinical treatment of gastrointestinal tumors, including Epothilone B, Sorafenib, Cisplatin, Doxorubicin, Etoposide, Imatinib, Lapatinib, OSI.906, PHA.665752, ABT.888, Camptothecin, Docetaxel, Mitomycin C, Paclitaxel, and Sunitinib. The half-maximal inhibitory concentration (IC50) of drugs was used to evaluate the therapy response of patients in two risk groups by R package “pRRophetic.” The significance threshold of all the above analyses results was *P* < 0.05 except for the multiple hypothesis test which used FDR to adjust.

### RNA extraction and RT-qPCR

The total RNA was extracted from serum samples using TRIzol LS Reagent (Invitrogen, Eugene, OR, USA). The concentration of RNA was measured using a NanoDrop spectrophotometer (Thermo Fisher Scientific, Waltham, MA, USA). RNA was reverse transcription into cDNA using SureScript RTase Mix and RT Reaction Buffer, and qPCR was performed using Blaze Taq qPCR Mix (GeneCopoeia, Guangzhou, China). The relative expression of target lncRNAs was normalized to the glyceraldehyde-3-phosphate dehydrogenase (GAPDH) and calculated 2 − ΔΔCt. The primer sequences are shown in Additional file 1: Table S1.

## Results

### Identification of CRLs and differentially expressed CRLs in COAD

**Fig. 1:**
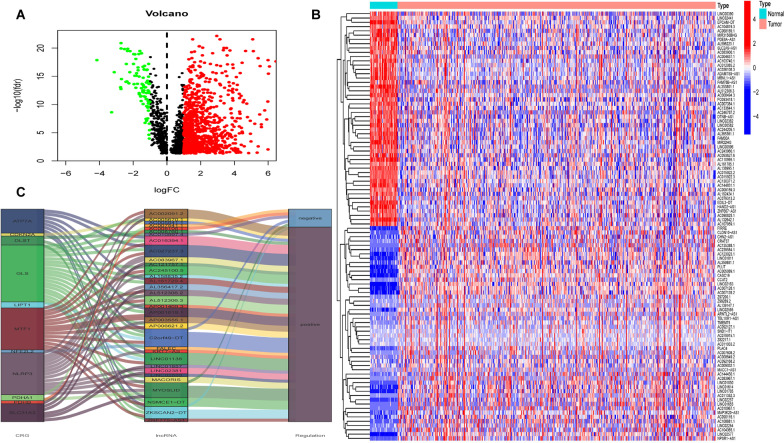
CRLs in COAD patients. **A** The volcano plot of CRLs, in which the red dots indicated upregulated lncRNAs in COAD tumors, the green dots indicated down-regulated lncRNAs, and black dots indicated lncRNAs that were no significant difference. **B** The heatmap of top 100 differentially expressed CRLs. **C** The Sankey diagram displayed the regulation of CRGs and CRLs

A total of 2003 lncRNAs were identified as CRLs according to the filters mentioned in the method section (Additional file 2: Table S2). The correlationship between CRGs and lncRNAs in COAD is shown in Additional file 3: Fig. S1. Then, the differential expression of CRLs between 473 COAD tumor and 41 normal tissues was compared and 1042 differentially expressed CRLs were obtained as shown in volcano plot (Fig. 1A, Additional file 4: Table S3). The top 100 CRLs were displayed by heatmap which could successfully separate tumor and non-tumor tissues (Fig. 1B).

### Establishment of the prognostic signature based on CRLs

**Fig. 2:**
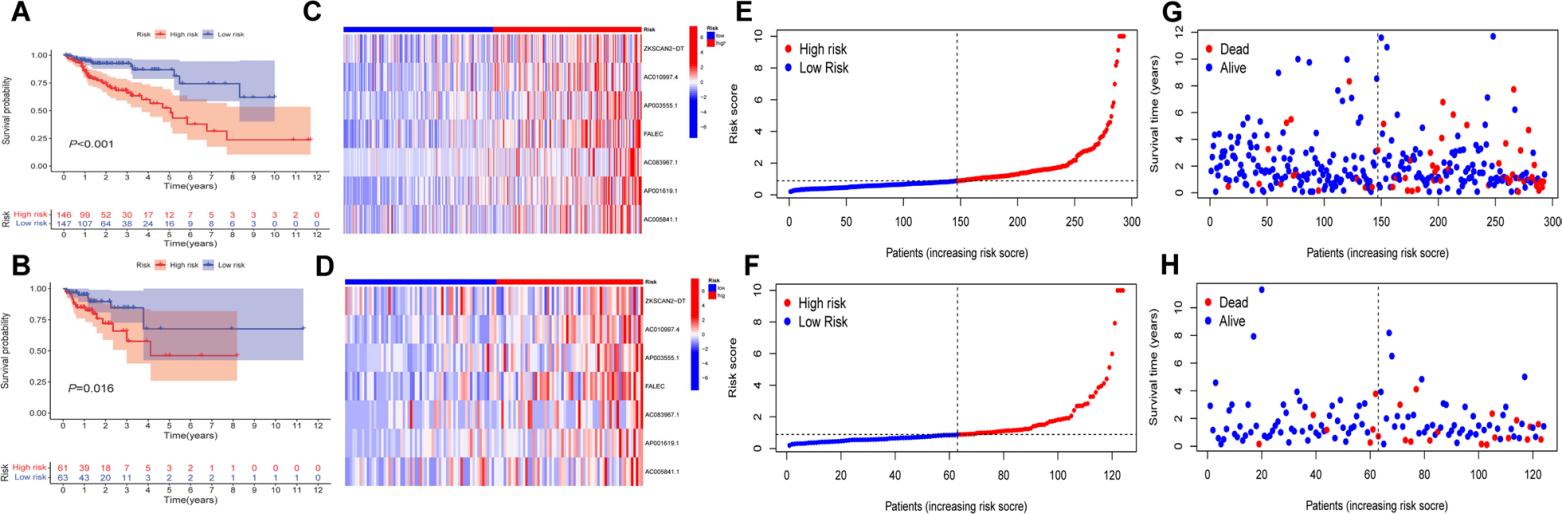
Establishment of prognostic signature based on CRLs in COAD. K–M curve for OS in training group (**A**) and test group (**B**), heatmap of 7 CRLs enrolled in the prognostic signature (**C, D**), the risk score curve (**E, F**) and survival status diagrams (**G**, **H**) of COAD patients in training group and test group

**Fig. 3:**
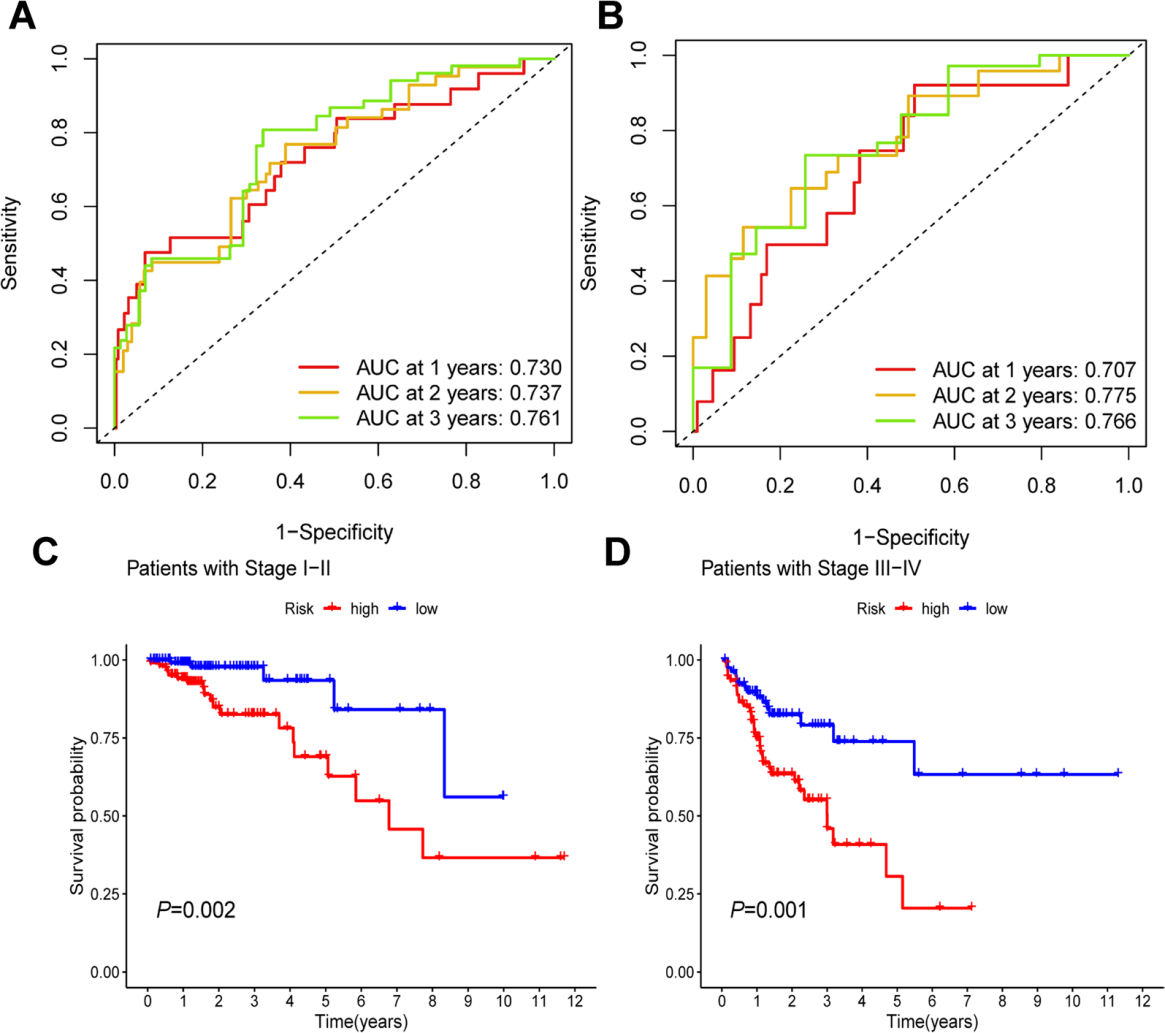
Evaluation of prognostic signature. The 1-, 2-, and 3-year ROC curves of prognostic signature in the training (**A**) and test (**B**) groups. K–M curve for OS of two risk groups in stage I–II patients (**C**) and stage III–IV patients (**D**)

We obtained 33 prognosis-related CRLs as shown in Additional file 5: Table S4. The Sankey diagram of CRGs and CRLs was plotted to display their correlationship in Fig. 1C. Then, we selected 7 CRLs to construct a prognostic signature by multivariate Cox regression analysis. The risk score was derived as follows: risk score = (1.817 × AC010997.4) + (0.727 × AP003555.1) + (1.03 × FALEC) + (0.908 × AC083967.1) + (0.822 × AC005841.1) + (1.366 × AP001619.1)—(0.767 × ZKSCAN2-DT). The patients were divided into high-risk and low-risk groups based on the median risk score. K–M curve showed that patients in the low-risk group had better outcomes than those in the high-risk group in both training and test groups (*P* < 0.05, Fig. 2A, B). Figure 2C–H illustrates the heatmap of the prognostic signature, distribution of risk score and survival status diagrams, respectively. The 1-, 2-, and 3-year AUC of the ROC curves in the training group was 0.73, 0.737, and 0.761, and that in the test group was 0.707, 0.775, and 0.766, respectively (Fig. 3A, B). The above results implied that the risk score performed well in predicting the prognosis of COAD patients. Besides, we also noticed that there was significant difference in both stage I–II (*P* = 0.002) and stage III–IV (*P* = 0.001) (Fig. 3C, D). So, our prognostic model was effective in predicting outcomes not only for advanced patients, but also for early patients in COAD.

**Fig. 4:**
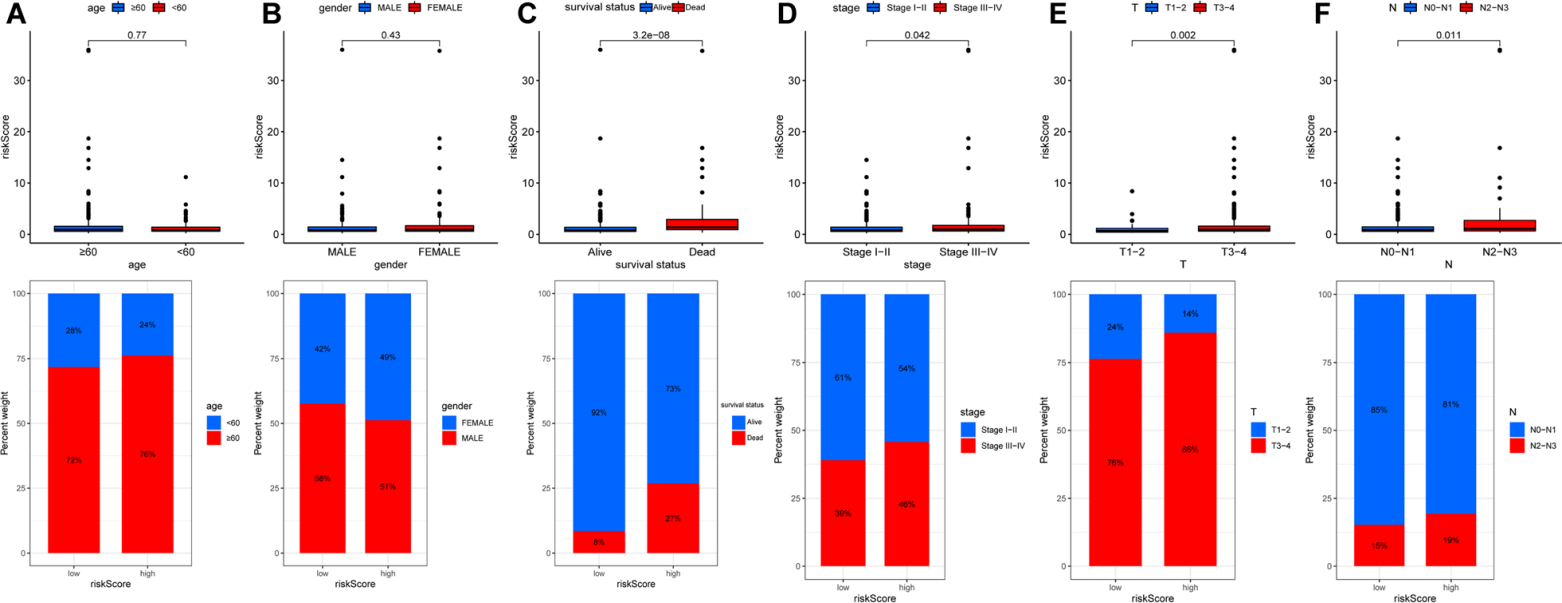
The relationship between the risk score and clinical parameters

The correlation between the risk score and clinical characteristics was analyzed. Our results demonstrated that the risk score was associated with survival status (*P* < 0.001), clinical stages (*P* = 0.042), T stage (*P* = 0.002) and N stage (*P* = 0.011) (Fig. 4C–F).

### Independent prognostic analysis and construction of a nomogram model

**Fig. 5:**
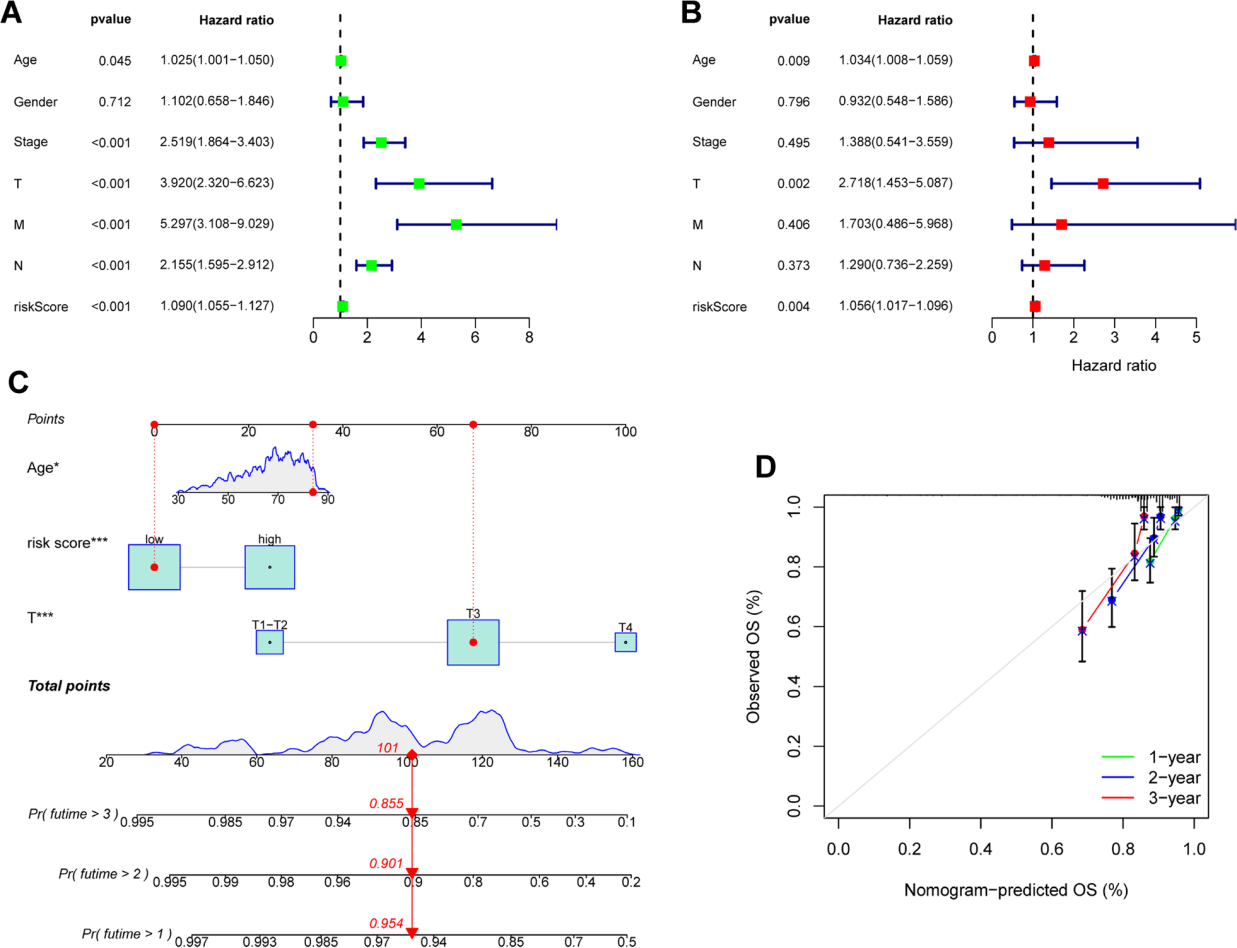
The nomogram model based on independent prognostic factors. Independent prognostic analysis by univariate Cox (**A**) and multivariate Cox regression (**B**). The nomogram model that integrated the risk score, age and T stage predicted the probability of the 1-, 2-, and 3-year OS (**C**) and the calibration plots (**D**)

By univariate and multivariate Cox regression analyses, we evaluated the independent predictors of COAD. The results showed that age (*P* = 0.045), clinical stage (*P* < 0.001), T stage (*P* < 0.001), N stage (*P* < 0.001), M stage (*P* < 0.001) and risk score (*P* < 0.001) were prognostic indicators (Fig. 5A). Furthermore, we found that only age (*P* = 0.009), T stage (*P* = 0.002) and risk score (*P* = 0.004) were independent prognostic indicators (Fig. 5B). Then, a nomogram model was constructed based on independent prognostic indicators which had excellent prognosis prediction ability (Fig. 5C). The accuracy of the nomogram model was estimated by the calibration plots which showed good consistency with the actual observation (Fig. 5D).

### KEGG pathways enrichment analysis by GSEA

**Fig. 6:**
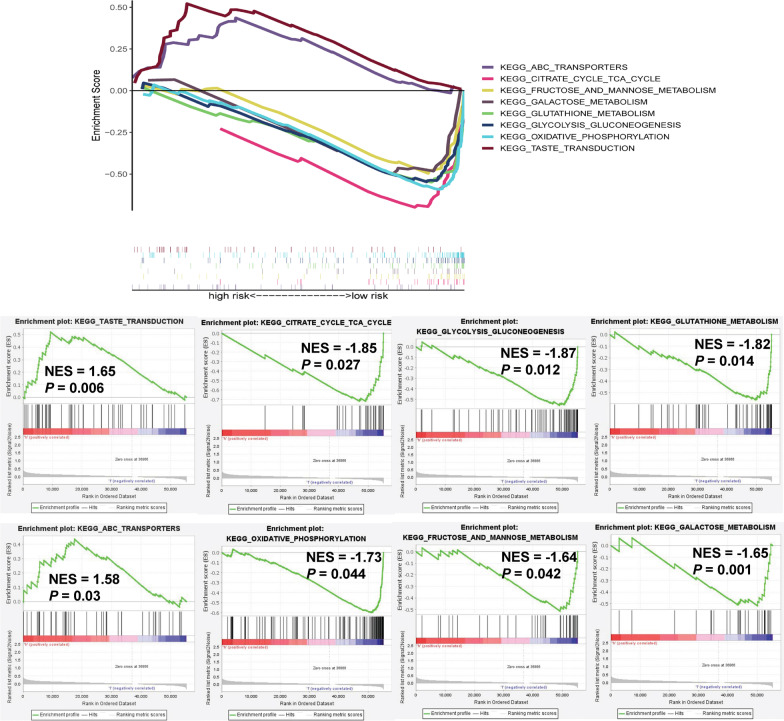
The KEGG signal pathway enriched analysis of high- and low-risk groups by GSEA

The results showed that enriched KEGG pathways had significant difference between high-risk group (Additional file 6: Table S5) and low-risk group (Additional file 7: Table S6). Interestingly, pathways of citrate cycle TCA cycle, glycolysis gluconeogenesis, oxidative phosphorylation, glutathione metabolism and so on were enriched in the low-risk group which might be the reason of different survival outcomes of two groups (Fig. 6).

### The difference of immune infiltration between two risk groups

**Fig. 7:**
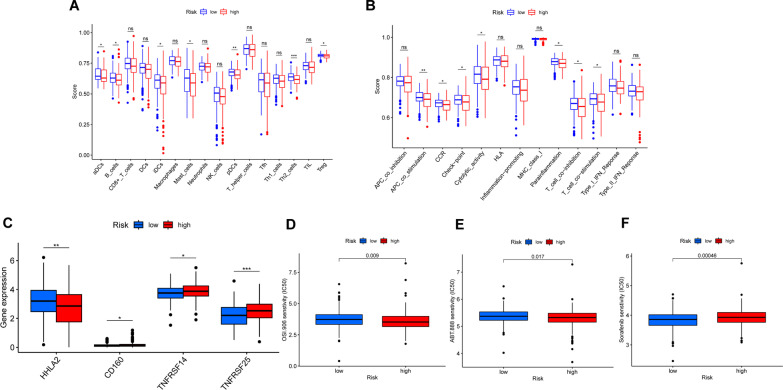
The investigation of tumor immune factors and drug sensitivity between two risk groups. The difference of immune cells (**A**), immune functions scores (**B**) and expression of immune checkpoints genes (**C**) between high- and low-risk groups. The drug sensitivity of two risk groups to OSI.906 (**D**), ABT.888 (**E**), Sorafenib (**F**). *FDR*: 0.05 > * > 0.01 > ** > 0.001 > ***

The immune scores of immune cells (aDCs, B cells, iDCs, Mast cells, pDCs, and so on.) and immune functions (APC co-stimulation, CCR, checkpoint, cytolytic activity, parainflammation, and so on) were higher in the low-risk group (Fig. 7A, B). The results showed that the risk score was negatively correlated with the infiltration of most anti-tumor immune cells.

### The relationship of risk score with immune checkpoints expression and drug treatment response

The expression of 4 immune checkpoints (HHLA2, CD160, TNFRSF14 and TNFRSF25) was different between two risk groups (Fig. 7C). Among them, the expression of HHLA2 was higher in the low-risk group. However, the expression of CD160, TNFRSF14 and TNFRSF25 was on the contrary. In the term of IC_50_ of 15 common drugs for gastrointestinal cancer, patients in the high-risk group were more sensitive to OSI.906, ABT.888 (Fig. 7D, E), while patients in the low-risk group were more sensitive to Sorafenib (Fig. 7F). The 95% CI of drug sensitivity analysis is shown in Additional file 8: Table S7.

### Differential expression of serum CRLs between COAD patients and healthy controls

**Fig. 8:**
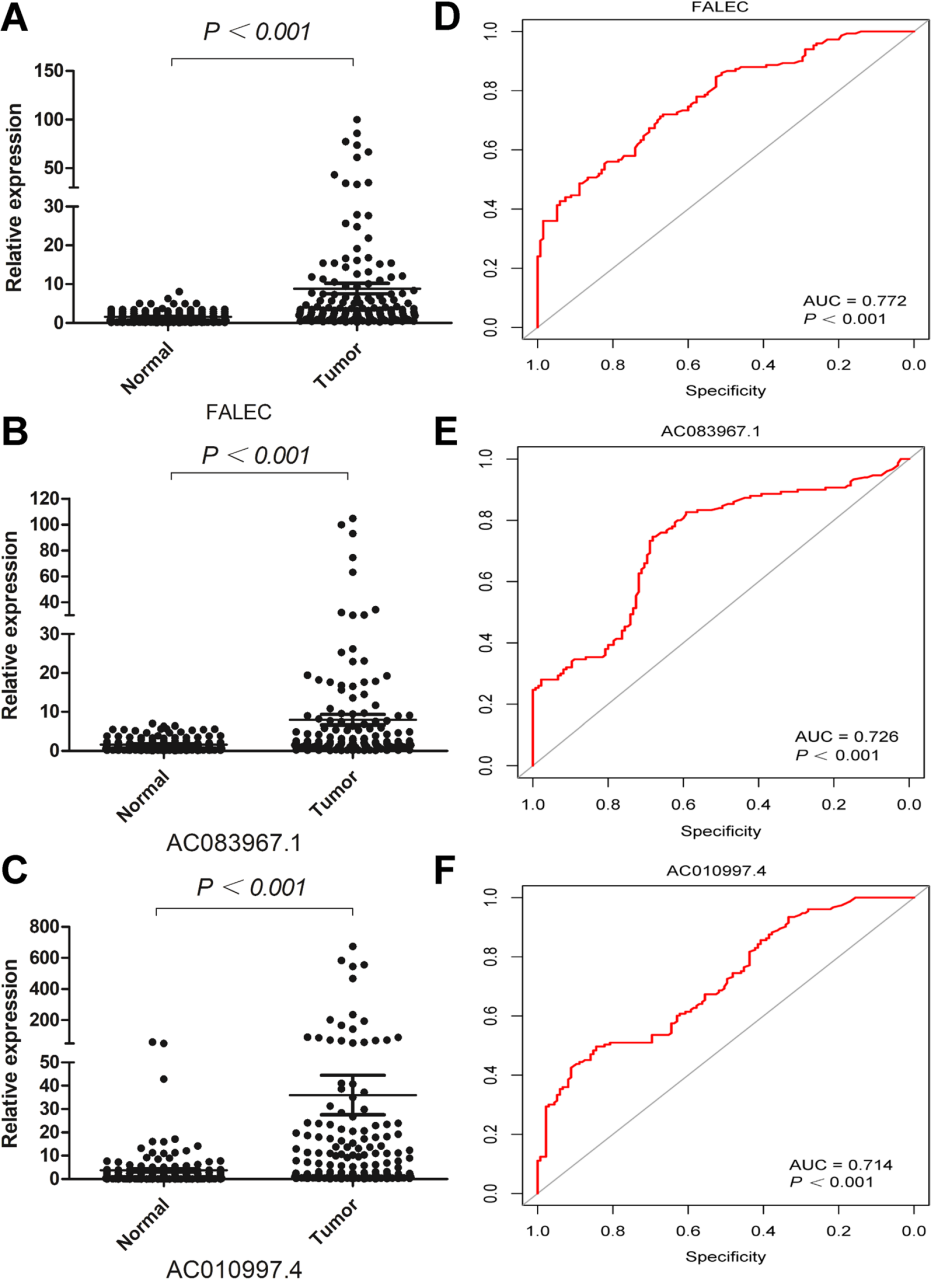
The expression of FALEC (**A**), AC083967.1 (**B**) and AC010997.4 (**C**) in serum of COAD patients and healthy controls by RT-qPCR. The ROC curves of FALEC (D), AC083967.1 (**E**) and AC010997.4 (**F**) to diagnosis COAD patients

To further explore the possible diagnostic values of CRLs, the expression of above 7 CRLs in serum between COAD patients and healthy controls was analyzed. Our results showed that three lncRNAs including FALEC, AC083967.1 and AC010997.4 were significantly increased in serum of COAD patients (Fig. 8A–C) which were consistent with RNA sequencing data of tissues. The AUC of FALEC, AC083967.1 and AC010997.4 was 0.772, 0.726 and 0.714, respectively, which indicated that they might be used as diagnostic markers (Fig. 8D–F). However, the other 4 CRLs were really low expressed in serum of both COAD patients and healthy controls which were hardly to accurately detected by RT-qPCR.

## Discussion

An appropriate amount of copper in cells is essential for life. It will impact the function of important metal binding enzymes if the content is too little, while too much of copper may lead to cell death [41]. Cuproptosis is a form of cell death caused by excessive copper that induces the aggregation of lipoylated dihydrolipoamide S-acetyltransferase (DLAT) and results in protein toxic stress [31, 42]. As critical regulators of gene expression, lncRNAs have the ability to affects the occurrence and development of cancers through multiple mechanisms including regulating gene–environment interaction of diseases [43, 44]. Studies have shown that epigallocatechin-3-gallate (EGCG) can regulate SLC31A1 (CTR1, copper transporter 1) expression through upregulated lncRNA nuclear, which effects cisplatin sensitivity for the treatment of non-small cell lung cancer cells [45]. Therefore, it is not surprising to speculate that related lncRNAs can regulate the process of cuproptosis to affect prognosis and treatment of COAD patients.

In the present study, we select 7 CRLs (AC010997.4, AP003555.1, FALEC, AC083967.1, AC005841.1, AP001619.1 and ZKSCAN2-DT) to establish a prognostic signature. Then, the K–M and ROC curves confirmed that the model had good predictive value for the prognosis of COAD patients. In fact, many lncRNAs in the signature have been proved to play the role in the prognosis of cancers. For example, FALEC can promote colorectal cancer progression via regulating miR-2116-3p-targeted PIWIL1 [46]. AC083967.1, AP001619.1 and AP003555.1 can be used as the prognostic marker for colorectal cancer [47–50]. The above studies are consistent with our results. Meanwhile, we also found that our prognostic signature was equally effective for stage I-II and stage III-IV of patients, which avoided the poor prediction of the prognosis for early COAD patients and increased the practicability of the signature. Besides, the risk score, age and T stage were identified as independent prognostic factors. So, we also built a nomogram model to comprehensively predict 1-year, 2-year, and 3-year OS of COAD patients based on independent factors. This is of great significance to evaluate the condition of COAD patients more accurately by combining risk score and clinical characteristics.

Tumor microenvironment (TME) that tumor cells depend on for growth and survival plays an important role in tumor progression. With the further understanding of the diversity and complexity of TME, its role in tumor progression, immune escape and immunotherapy response has been paid more and more attention [51]. By GSEA and ssGSEA analyses, we found that patients in the high-risk group had a significant low degree of immune infiltration. We believed that these patients had obvious immunosuppression and belonged to immune-desert phenotype which was characterized by immune tolerance, immune ignorance and associated with poor clinical outcomes [52]. Interestingly, patients in the low-risk group were enriched in glucose-related metabolic pathways, which were closely related to cuproptosis and had high level of immune cell infiltration and immune function. Therefore, we speculated that these patients were immune-inflamed phenotype which was characterized by adaptive immune cell infiltration and immune activation [53–55]. Also, cuproptosis might inhibit the progression of COAD partly, which requires further research to explore the potential mechanism.

Immunotherapy represented by immune checkpoint blockade (ICB) has shown amazing clinical efficacy and durable responses. But it is only for a small number of cancer patients, and most patients have little benefit, far from a met clinical need [56]. Analyzing the expression of immune checkpoint genes in COAD patients will help to improve the effect of immunotherapy. We found that the expression of CD160, TNFRSF14, TNFRSF25 was higher in high-risk group, yet the expression of HHLA2 was higher in low-risk group. Therefore, ICBs for the above genes could implement more accurate treatment for different patients. Besides, we also collected 15 commonly used drugs for gastrointestinal tumors and analyzed the sensitivity differences between two risk groups. We found that patients in the high-risk group were more sensitive to OSI.906 and ABT.888, while patients in the low-risk group were more sensitive to Sorafenib. These results could provide further guidance for the personalized drug treatment of patients with COAD based on our signature.

To further explore whether the above 7 CRLs also have diagnostic value, we detected the expression of CRLs in serum by RT-qPCR. Compared with healthy controls, FALEC, AC083967.1 and AC010997.4 were highly expressed in serum of COAD patients and ROC curves also verified their good diagnostic value. Interestingly, the diagnostic value of plasma FALEC in cervical cancer has been reported and considered as a promising diagnostic marker [57].

## Conclusions

Overall, although our prognostic signature performed well in predicting the diagnosis, prognosis and treatment responses of COAD, our current study inevitably had some limitations. On the one hand, it is hard for our team to follow-up large amount of COAD patients to acquire survival information and verify the signature. On the other hand, the research on cuproptosis is still in the early stage at present, and the fewer CRGs limit the scope of research. Our results provided new insights into understanding the relationship of COAD tumorigenesis mechanism and cuproptosis, and further molecular and clinical trials are still needed to confirm our findings. We believe that with the further research on cuproptosis in the future, it will have more comprehensive and in-depth understanding to improve relevant research.

## Supplementary Information

**Additional file 1: Table S1.** The primer sequences of CRLs

**Additional file 2: Table S2.** The cuproptosis-related lncRNAs

**Additional file 3: Fig. S1.** The network between CRGs and CRLs

**Additional file 4: Table 3.** The differential expressed CRLs between COAD tumor and normal tissues

**Additional file 5: Table 4.** The 33 prognosis-related CRLs about COAD

**Additional file 6: Table 5.** The results of GSEA analysis in high-risk group

**Additional file 7: Table 6.** The results of GSEA analysis in low-risk group

**Additional file 8: Table 7.** The 95 % CI of drug sensitivity analyzed by Wilcox test

## Data Availability

Statistical analyses were performed using the R 4.1.2 software, Strawberry Perl (64-bit) 5.30.0.1, GSEA 4.2.2. The Wilcox test was used to compare difference between COAD tumors and normal tissues, serum expression of CRLs between COAD patients and healthy controls and differences of immune cells, immune functions between two risk groups. Chi-squared test was used to perform K–M survival analysis of the prognostic model. Differences were considered statistically significant when *P* value or FDR was < 0.05. All R scripts and data generated or analyzed during this study are available from the corresponding author on reasonable request.

## Abbreviations

CRGs: Cuproptosis-related genes
CRLs: Cuproptosis-related lncRNAs
lncRNAs: Long noncoding RNAs
CRC: Colorectal cancer
CC: Colon cancer
RC: Rectal cancer
COAD: Colon adenocarcinoma
TCGA: The Cancer Genome Atlas
