# Supplementary figures and images for: Comprehensive analysis of cuproptosis-related long noncoding RNA for predicting prognostic and diagnostic value and immune landscape in colorectal adenocarcinoma
Source: Hum Genomics. 2023 Mar 13;17:22. doi: 10.1186/s40246-023-00469-5 (PMC10009981; doi:10.1186/s40246-023-00469-5)

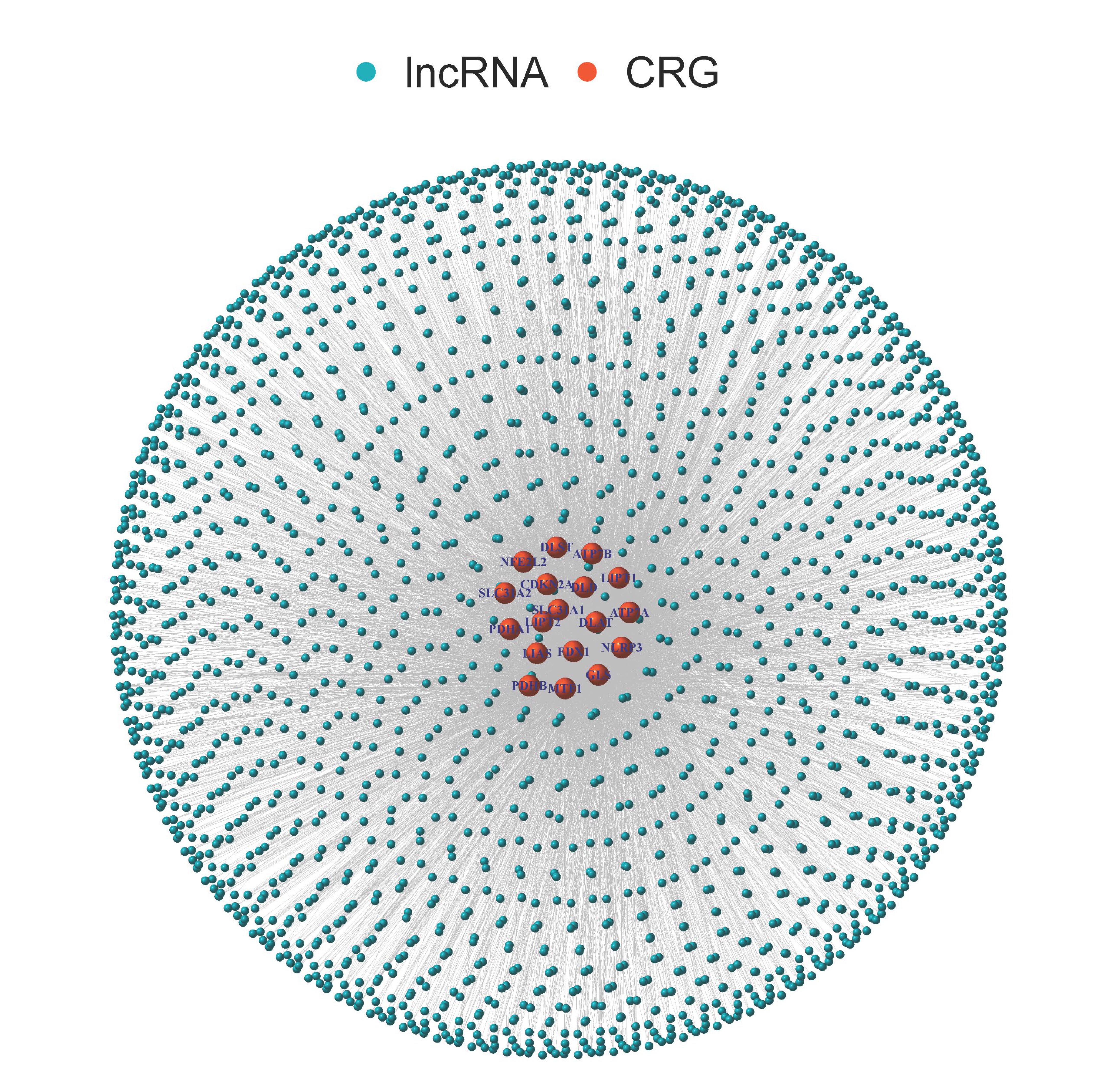

Supplement: Supplementary file 3 — Additional file 3: Fig. S1. The network between CRGs and CRLs [file 40246_2023_469_MOESM3_ESM.tif]
